# Development and validation of a nomogram for predicting the risk of postoperative fracture blister after pilon fracture

**DOI:** 10.3389/fsurg.2024.1401087

**Published:** 2024-10-10

**Authors:** Peiyuan Wang, Chengsi Li, Lin Liu, Zihang Zhao, Zhiang Zhang, Kuo Zhao, Wei Chen, Yingze Zhang, Lin Jin, Zhiyong Hou

**Affiliations:** ^1^ Orthopaedic Research Institute of Hebei Province, The Third Hospital of Hebei Medical University, Shijiazhuang, Hebei, China; ^2^ Department of Orthopaedic Surgery, The Third Hospital of Hebei Medical University, Shijiazhuang, Hebei, China; ^3^ NHC Key Laboratory of Intelligent Orthopaedic Equipment, The Third Hospital of Hebei Medical University, Shijiazhuang, China

**Keywords:** fracture blister, pilon fractures, postoperation, body mass index, neutrophil, creatine kinase (MB form), plate fixation, the mode of surgical suture

## Abstract

**Background:**

Fracture blister (FB) is one of the most common complications in pilon fractures. This study aimed to construct and validate a nomogram for predicting postoperative FB risk in patients with pilon fractures.

**Methods:**

We retrospectively collected information on 1,119 patients with lower extremity fractures in the 3rd Hospital of Hebei Medical University between January 2023 and January 2024. Patients with FBs were considered as the FB group and those without FB as the non-FB group. Variables with a significance level of *P* < 0.05 in the univariate analysis were included in the multivariate logistic regression analysis. The backward stepwise regression method was applied to identify independent risk factors associated with FB. The selected predictors were then entered into R software for further analysis and Nomogram construction.

**Results:**

In our research, the rate of FB (119 of 1,119) was 10.63%. Several predictors of FB were found using univariate analysis, including body mass index (BMI) (*p* < 0.001), the presence of DVT (*p* < 0.001), closed fractures (*p* < 0.001), time from injury to admission (*p* < 0.001), smoking history (*p* < 0.01), not utilizing dehydrating agents (*p* < 0.010), fixation mode of fracture (*p* < 0.001), the mode of surgical suture (*p* < 0.001), postoperative infection (*p* < 0.001) and Elixhauser comorbidity index (ECI) (*p* < 0.01). In addition, FB group exhibited significantly higher levels of blood serum indicators, such as EOS (*p* = 0.029), HCT (*p* < 0.01), LYM (*p* = 0.01), MPV (*p* = 0.014), NEU (*p* < 0.01), CKMB (*p* < 0.01), PLT (*p* < 0.01), ALB (*p* < 0.01), ALP (*p* < 0.01), AST (*p* < 0.01), CK (*p* = 0.019), CREA(*p* < 0.01), DBIL (*p* < 0.01), GLU (*p* < 0.01), Na (*p* < 0.01), P (*p* < 0.01), TC (*p* = 0.024), ALT (*p* < 0.01), TCO_2_ (*p* < 0.01), TG (*p* < 0.01), TP (*p* < 0.01), UA (*p* = 0.018), UREA (*p* = 0.033) compared to the non-FB group. According to the stepwise logistic regression analysis, higher BMI (*p* = 0.011, OR 0.873, 95% CI 0.785–0.970), NEU (*p* = 0.036, OR 0.982, 95% CI 0.865–0.995) and CKMB (*p* < 0.014, OR 0.994, 95% CI 0.989–0.999) were associated with increased FB risk, while plate fixation (*p* = 0.017, OR 0.371, 95% CI 0.123–0.817), the mode of surgical suture (*p* < 0.01, OR 0.348, 95% CI 0.161–0.749), and postoperative infection (*p* = 0.020, OR 0.406, 95% CI 0.190–0.866) were also correlated with increased FB risk. The nomogram was established based on 6 predictors independently related to FB.

**Conclusions:**

Our investigation has shown that BMI, NEU, CKMB, plate fixation, the mode of surgical suture, and postoperative infection are independent risk factors for FB in patients with pilon fractures. The predictors identified by the nomogram could potentially be used to assess the possibility of blister formation, which could be a sign of fascial compartmental pressure release.

## Introduction

The term “pilon fracture” lacks a rigorously defined delineation, but it typically denotes a fracture occurring in the distal third of the tibia, encompassing the articulating surface of the distal tibia. These fractures represent significant intra-articular injuries to the distal tibia, predominantly caused by substantial axial loading. Concurrent soft tissue trauma, frequently evidenced by the formation of fracture blisters, accompanies these high-energy injuries, heightening the likelihood of wound complications and infection in affected patients (1). In existing research, wound healing complication rates in pilon fractures have reportedly ranged from 9% to 18% (1–3). This condition typically manifests in the distal tibia due to low-energy twisting or high-energy axial forces resulting from falls from a height or motor vehicle accidents. As such, injuries may manifest in the articular and metaphyseal regions of the tibia, either independently or concomitantly with fibular fracture (4–11). Such fractures often impact a substantial portion of the weight-bearing articular surface and, owing to the extensive involvement of the ankle joint, pose challenges in respect of treatment and fixation (12).

Meanwhile, FBs are predominantly noted in pilon fractures, calcaneal fractures, and ankle fractures, which typically occur in regions of comparatively fragile skin, such as the ankle, hind foot, and proximal tibia (13, 14). In 1986, Shelton and Anderson described fracture blisters as “areas of epidermal necrosis with a separation of the stratified squamous cell layer from the underlying vascular dermal layer by edema fluid” (15). A revised conceptualization of fracture blisters was introduced by Varela et al. (16) in 1993, who defined them as “tense vesicles or bullae arising in swollen skin over a fracture”. To comprehend fracture blisters, it is essential to grasp two fundamental principles: firstly, the molecular harm inflicted upon soft tissue injury; and secondly, the subsequent formation of blisters as a consequence of this molecular damage (13). In simpler terms, fracture blisters are a relatively rare complication that occurs acutely with a low incidence rate of 2.9%. Typically, blisters appear within 24 h of the initial injury, although they can appear as early as six hours later (16, 17). In terms of appearance and histology, FBs can be classified as clear fluid-filled or hemorrhagic blisters (14, 18–20). Upon blister formation, the structural integrity of the skin becomes compromised, increasing the likelihood of infection. Accordingly, medical practitioners may need to modify their initial treatment strategies. Strauss et al. concluded that blood-blisters are potentially associated with a higher rate of complications (14, 21). Further, they noted that it is generally safe to make incisions around intact blisters, and clear-filled blisters can also be incised. However, it is not advisable to incise blood-filled blisters due to the higher risk of infection they pose (14, 21). Moreover, blisters were confirmed as a major factor in delaying definitive treatment (14, 21). Hou's team conducted an in-depth study of fracture blisters. Their findings suggested that these blisters might signify a decrease in intrafascial pressure following traumatic injury. This pressure decrease can prevent the harmful cycle of ischemia and hypoxia triggered by further elevation of intrafascial pressure, thus averting the development of severe intrafascial hypertension (22–24).

Nevertheless, there is a scarcity of research that has focused on FBs after the surgery of pilon fractures. Although FBs can potentially lead to complications, they have been demonstrated to serve as a favorable indicator of the release of intracompartmental pressure, thereby preventing further escalation of pressure. In order to identify risk factors associated with the formation of postoperative FBs, a retrospective review of patients with pilon fractures was conducted.

## Materials and methods

### Ethics statement

An analysis of all electronic medical records of patients at the 3rd Hospital Hebei Medical University treated for pilon fractures from January 2023 to January 2024 were conducted. Ethical clearance was secured from the institutional review boards of the hospital (Ethic approval number: NCT04529330, S2020-022-1), aligning with the ethical principles outlined in the Helsinki Declaration of 1964.

### General information

**Figure 1:**
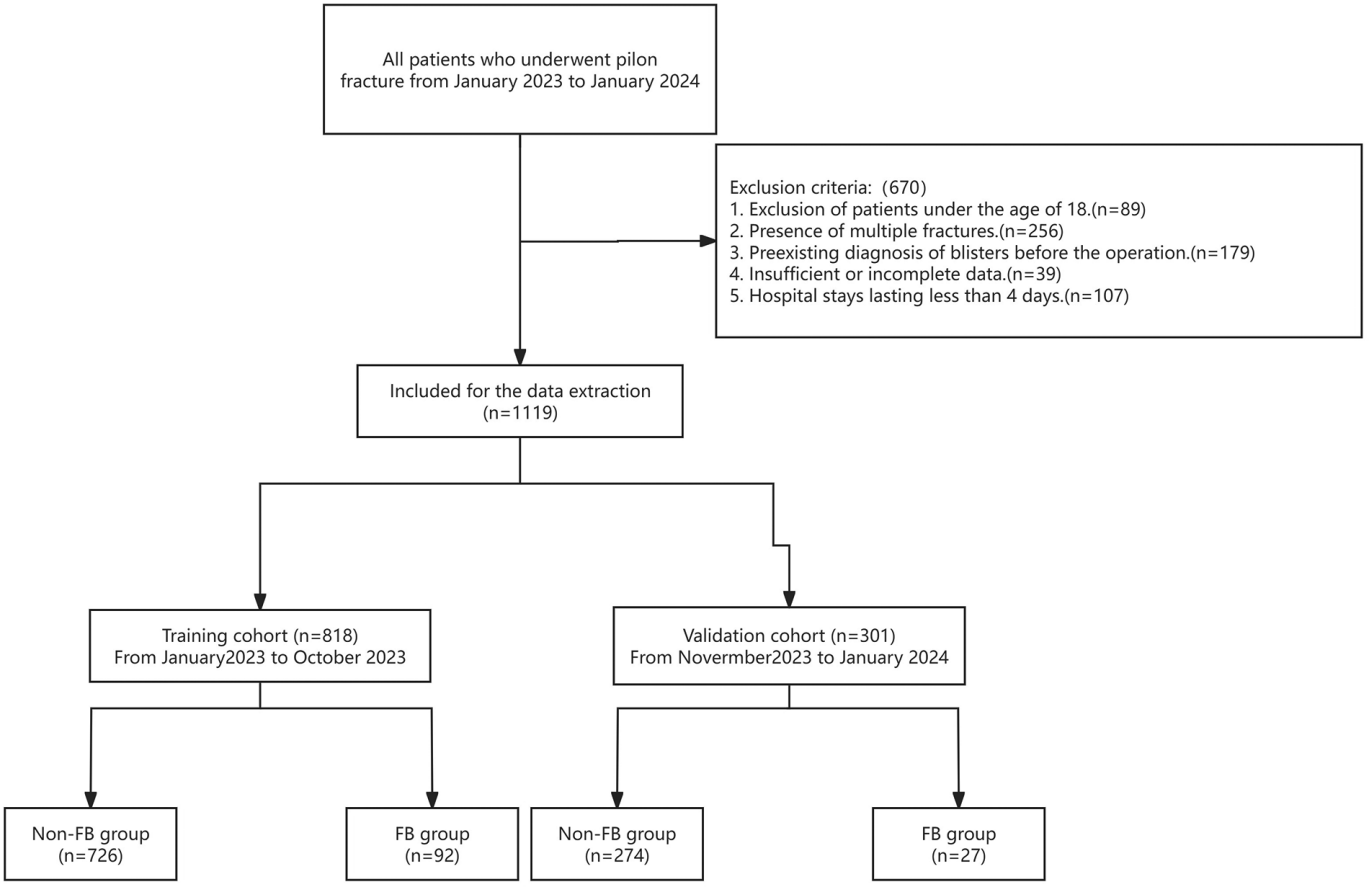
Patients selection flowchart.

The aim of the present retrospective study was to gather clinical data from patients diagnosed with pilon fractures who were admitted to our hospital between January 2023 and January 2024. The inclusion criteria were as follows: (1) Enrollment of patients aged 18 years or older; and (2) Verification of pilon fractures through x-ray examination. Conversely, the exclusion criteria encompassed: (1) Exclusion of patients under the age of 18; (2) Presence of multiple fractures; (3) Pre-existing diagnosis of blisters before the operation; (4) Insufficient or incomplete data; and (5) Hospital stays lasting less than 4 days (Figure 1).

### Methods

Data on patient demographics, comorbidities, and preoperative laboratory examinations was systematically collected. The demographic variables encompassed age, gender, body mass index (BMI), fracture types, deep vein thrombosis (DVT), Elixhauser comorbidity index (ECI), smoking history, alcohol consumption, injury mechanism, time from injury to admission, use of a dehydrating agent, intraoperative fixation, intraoperative suture, and postoperative infection.

Additionally, a thorough investigation was conducted to assess various preoperative laboratory indicators, including albumin (ALB), alkaline phosphatase (ALP), aspartate aminotransferase (AST), alanine transaminase (ALT), calcium (Ca), potassium (K), sodium (Na), magnesium (Mg), phosphorus (P), chloride (Cl), globulin (GLOB), cholinesterase (CHE), creatine kinase (CK), creatinine (CREA), direct bilirubin (DBIL), glucose (GLU), lactic dehydrogenase (LDH), triglyceride (TG), total cholesterol (TC), total carbon dioxide (TCO_2_), ureophil (UREA), uric acid (UA), basophil (BAS), eosinophil (EOS), hematocrit (HCT), hemoglobin (HGB), lymphocyte (LYM), mean corpuscular hemoglobin concentration (MCHC), monocyte (MON), mean platelet volume (MPV), neutrophil (NEU), creatine kinase isoenzyme (CKMB), platelet (PLT), red blood cell (RBC), and white blood cell (WBC).

### Routine management of patients with postoperative pilon fracture

Antibiotics were used 1 h before surgery and 24 h after surgery to prevent infection (cefazolin sodium was routinely used, with clindamycin employed for patients with cephalosporin allergies), and oral rivaroxaban or subcutaneous injection of low molecular weight heparin to prevent DVT 48 h after surgery (in cases of DVT, the anticoagulant dose was escalated from prophylactic to therapeutic levels). Blood indexes in the present study were obtained from the initial blood collection conducted after surgery.

### Statistical analysis

In the present study, SPSS software (version 25.0, SPSS Inc., New York, USA) was utilized, and *p* < 0.05 was considered statistically significant. The normality of continuous variables was assessed using the Shapiro-Wilk test. If normality was met, these variables were typically expressed as mean (range) and analyzed using the *t*-test. In instances where the assumption of normality was not satisfied, the Mann-Whitney *U*-test was utilized. For categorical variables, which were represented by numerical counts and percentages, statistical analysis was conducted using the Chi-square test and Fisher's exact test to evaluate differences between groups.

Variables with a significance level of *P* < 0.05 in the univariate analysis were included in the multivariate logistic regression analysis. The backward stepwise regression method was employed to identify independent risk factors associated with FB. The selected predictors were then inputted into R software (Version 3.6.5, R Foundation for Statistical Computing, Vienna, Austria) for further analysis. The “rms” package was employed to develop the nomogram.

The predictive power and application value of the model were evaluated using a range of assessment metrics, which included the Conformance index (C-index), receiver operating characteristic (ROC) curve, calibration curve, and decision curve analysis (DCA). The C-index, which ranged from 0.5 to 1.0, along with the area under the receiver operating characteristic curve (AUC), were positively correlated with the discriminative capacity and predictive accuracy of the model, as illustrated by the nomogram.

Calibration curves were employed to assess the concordance between diagnosed FB and predicted FB. A greater alignment between the predicted curve and the ideal curve indicated a stronger correspondence between the predicted probability and the actual probability. The clinical utility of the nomogram model was evaluated using DCA, which integrates threshold probabilities and net benefit to evaluate its practical application. To enhance the validation of the model's prediction performance, the Bootstrap method was employed for internal verification, and the modified C-index was computed through 1,000 iterations of sampling. Then we employed the validation set for further external verifcation and compared the performances of ROC curves, calibration curves, and DCA of the nomogram in the training and validation cohorts. A significance level of 0.05 was applied to all analyses.

## Results

A total of 1,789 cases were reviewed, with 670 cases being excluded from the study. A total of 1,119 cases met the study criteria with 989 males and 130 females. The incidence of FB in patients with postoperative pilon fractures was 10.63%, affecting 119 patients and leaving 1,000 unaffected. The validation cohort includes 301 cases, at the same time, the training cohort contains 818 cases, which presented in Table 1. The statistical analysis indicated significant differences between the FB and non-FB groups with respect to several variables, including BMI (*p* < 0.001), the presence of DVT (*p* < 0.001), closed fractures (*p* < 0.001), time from injury to admission (*p* < 0.001), smoking history (*p* < 0.01), the administration of dehydrating agents (*p* < 0.010), fixation mode of fracture (*p* < 0.001), the mode of surgical suture (*p* < 0.01), postoperative infection (*p* < 0.01), and ECI (*p* < 0.01). Specifically, individuals in the FB group demonstrated higher BMI levels compared to those in the non-FB group. Moreover, in contrast to the non-FB group, patients in the FB group were more susceptible to developing DVT or sustaining closed fractures. Additionally, the use of dehydrating agents was associated with a decreased likelihood of developing FBs. Further, individuals in the FB group were more likely to have a history of smoking. Meanwhile, patients who underwent plate fixation or conventional suture techniques were more prone to developing FBs, whereas the non-FB group experienced fewer postoperative infections. Finally, the FB group possessed a higher ECI. In contrast, there were no statistically significant differences observed between the two groups regarding age, gender, mechanism of injury, or history of alcohol consumption (all *p* > 0.05).

**Table 1:** General information related to FB in patients.

Variable	Non FB group (*n* = 726)	FB group (*n* = 92)	*P*
Gender, *n* (%)			0.778
Female	78 (10.74%)	9 (9.78%)	
Male	648 (89.26%)	83 (90.22%)	
Age (years)	42.0 (27.0–59.0)	38.0 (29.0–45.0)	0.320
BMI (kg/m^2^)	24.1 (22.3–26.0)	26.5 (25.3–27.3)	<0.01
DVT, *n* (%)			<0.01
No	645 (88.84%)	55 (59.78%)	
Yes	81 (11.16%)	37 (40.22%)	
Open fracture, *n* (%)			<0.01
No	288 (39.67%)	60 (65.22%)	
Yes	438 (60.33%)	32 (34.78%)	
Mechanism of injury, *n* (%)			0.835
Fall down	228 (31.40%)	27 (29.35%)	
Traffic injury	252 (34.71%)	31 (33.70%)	
High falling	246 (33.88%)	34 (36.96%)	
Time from injury to admission, *n* (%)			<0.01
<1	450 (61.98%)	18 (19.57%)	
1–6	174 (23.97%)	16 (17.39%)	
≥7	102 (14.05%)	58 (63.04%)	
Alcohol history, *n* (%)			0.691
No	222 (30.58%)	30 (32.61%)	
Yes	504 (69.42%)	62 (67.39%)	
Smoking history, *n* (%)			<0.01
No	486 (66.94%)	40 (43.48%)	
Yes	240 (33.06%)	52 (56.52%)	
Dehydrating agent, *n* (%)			<0.01
No	264 (36.36%)	62 (67.39%)	
Yes	462 (63.64%)	30 (32.61%)	
AO classification, *n* (%)			0.051
43A	257 (35.40%)	21 (22.83%)	
43B	326 (44.90%)	25 (27.17%)	
43C	143 (19.70%)	46 (50.00%)	
Fixation mode of fracture, *n* (%)			<0.01
External fixation	499 (68.7%)	14 (15.2%)	
Intramedullary nail	121 (16.7%)	13 (14.1%)	
Plate fixation	106 (14.6%)	65 (70.7%)	
The mode of surgical suture, *n* (%)			<0.01
De-tensioning suture	619 (85.2%)	28 (30.4%)	
Conventional suture	107 (14.8%)	64 (69.6%)	
Postoperative infection, *n* (%)			<0.01
No	610 (84.0%)	25 (27.1%)	
Yes	116 (16.0%)	67 (72.9%)	
ECI	4.0 (2.0–4.0)	8.0 (5.0–9.0)	<0.01

A comparison of laboratory test results between the two groups is outlined in Table 2. Compared to the non-FB group, the FB group exhibited significantly higher levels of HCT (*p* < 0.01), MPV (*p* = 0.014), NEU (*p* < 0.01), CKMB (*p* < 0.01), ALB (*p* < 0.01), AST (*p* < 0.01), CK (*p* = 0.019), GLU (*p* < 0.01), TG (*p* < 0.01), and TP (*p* < 0.01). Conversely, EOS (*p* = 0.029), LYM (*p* < 0.01), PLT (*p* < 0.01), ALP (*p* < 0.01), CERA (*p* < 0.01), DBIL (*p* < 0.01), Na (*p* < 0.01), *P* (<0.01), TC (*p* = 0.024), ALT (*p* < 0.01), TCO_2_ (*p* < 0.01), UA (*p* = 0.018) and UERA (*p* = 0.033) in the FB group were lower compared to the non-FB group. However, no significant differences were observed in other laboratory data between these two groups (all *p* > 0.05).

**Table 2:** Laboratory results of patients.

Variable	Non FB group (*n* = 726)	FB group (*n* = 66)	*P*
BAS	0.04 (0.01–0.05)	0.04 (0.02–0.05)	0.92
EOS	0.14 (0.07–0.15)	0.14 (0.01–0.16)	0.029
HCT	36.18 (34–40.39)	36.06 (33.32–37.76)	<0.01
HGB	122.19 (113.05–136.87)	121.67 (112.86–130.96)	0.964
LYM	1.64 (1.23–1.77)	1.58 (1.142–1.64)	<0.01
MON	1.06 (0.72–1.07)	1.06 (0.8–1.12)	0.778
MPV	8.87 (8.17–9.11)	8.83 (8.18–9.48)	0.014
NEU	8.3 (7.4–11.6)	141 (12.9–15.1)	<0.01
CKMB	43.1 (24.125–114.35)	97.849 (68.64–107.57)	<0.01
PLT	230.51 (182.007–251.59)	175.935 (160.178–190.942)	<0.01
RBC	4.49 (3.68–7.89)	4.43 (3.532–6.81)	0.175
WBC	15.51 (11.92–16.8)	15.41 (10.79–19.66)	0.058
ALB	34.28 (33.01–39.4)	34.8 (33.06–35.71)	<0.01
ALP	73.01 (54.05–73.8)	70.64 (63.94–71.21)	0.001
AST	50.05 (28.01–63.03)	51.85 (29.69–52.34)	<0.01
Ca	64.05 (30–142.42)	103.84 (32.1–105.28)	0.099
CHE	2.06 (1.98–2.2)	2.08 (1.97–2.18)	0.343
CK	6.38 (5.48–6.81)	6.56 (5.76–6.98)	0.019
CL	1,701.01 (387.67–5,188.53)	3,758.28 (417.75–3,758.37)	0.398
CREA	104.08 (102.81–106.21)	104.38 (100.21–104.5)	<0.01
DBIL	72.63 (60.77–73.58)	69.87 (67.682–74.1)	<0.01
GLOB	5.23 (3.37–6.1)	5.27 (4.195–5.49)	0.413
GLU	21.83 (19.23–22.43)	22.04 (22.02–24.79)	<0.01
K	7.95 (6.32–7.99)	8.03 (6.732–8.17)	0.994
LDH	4.03 (3.68–4.13)	3.95 (3.71–4.17)	0.352
Mg	753.01 (276.5–933.92)	735.98 (416.473–767.92)	0.587
Na	0.83 (0.77–0.94)	0.81 (0.78–0.88)	<0.01
P	137.22 (136.01–139.31)	137.78 (134.91–138.92)	<0.01
TC	1.18 (0.98–1.24)	1.13 (1.08–1.2)	0.024
ALT	3.25 (2.77–3.69)	3.31 (2.63–3.34)	<0.01
TCO2	23.54 (22.02–25.3)	23.42 (22.46–23.44)	<0.01
TG	1.38 (0.82–1.39)	1.38 (1.01–1.39)	<0.01
TP	56.15 (52.05–62.33)	56.83 (53.928–63.11)	0.005
UA	332.39 (261.05–356.93)	325.84 (281.65–325.88)	0.018
UREA	5.69 (4.51–5.8)	5.38 (4.41–5.75)	0.033

The stepwise regression logistic analysis identified significant factors related to FB occurrence. Higher BMI (*p* = 0.011, OR 0.873, 95% CI 0.785–0.970), NEU (*p* = 0.036, OR 0.982, 95% CI 0.865–0.995) and CKMB (*p* < 0.014, OR 0.994, 95% CI 0.989–0.999) were associated with increased FB risk, while intramedullary nail (*p* = 0.017, OR 0.371, 95% CI 0.123–0.817), de-tensioning suture (*p* < 0.01, OR 0.348, 95% CI 0.161–0.749), and postoperative infection (*p* = 0.020, OR 0.406, 95% CI 0.190–0.866) were also correlated with increased FB risk. Further, as presented in Table 3, no additional factors influencing the incidence of FBs in these patients were identified.

**Table 3:** Binary logistic regression analysis of variables associated with FB.

Binary logistic regression analysis of variables associated with FB			
Characteristics	OR	95% CI	*P*
BMI	0.873	0.785–0.97	0.011
NEU	0.928	0.865–0.995	0.036
CKMB	0.994	0.989–0.999	0.014
Intramedullary nail	0.515	0.217–1.223	0.133
Plate fixation	0.317	0.123–0.817	0.017
The mode of surgical suture	0.348	0.161–0.749	<0.01
Postoperative infection	0.406	0.19–0.866	0.020

**Figure 2:**
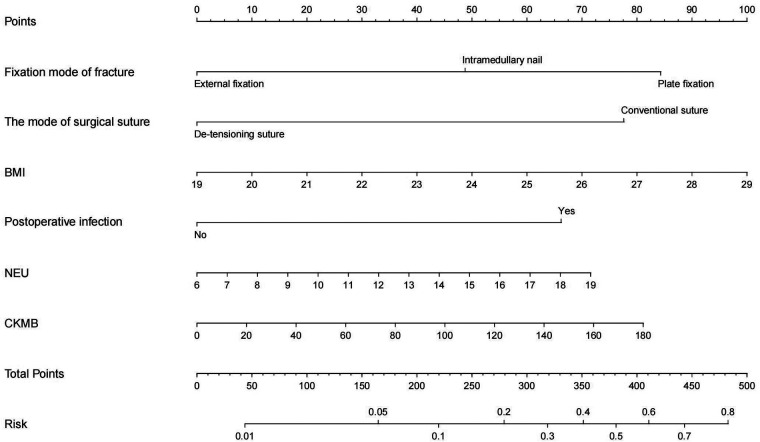
The nomogram designed for predicting the risk of FB.

**Figure 3:**
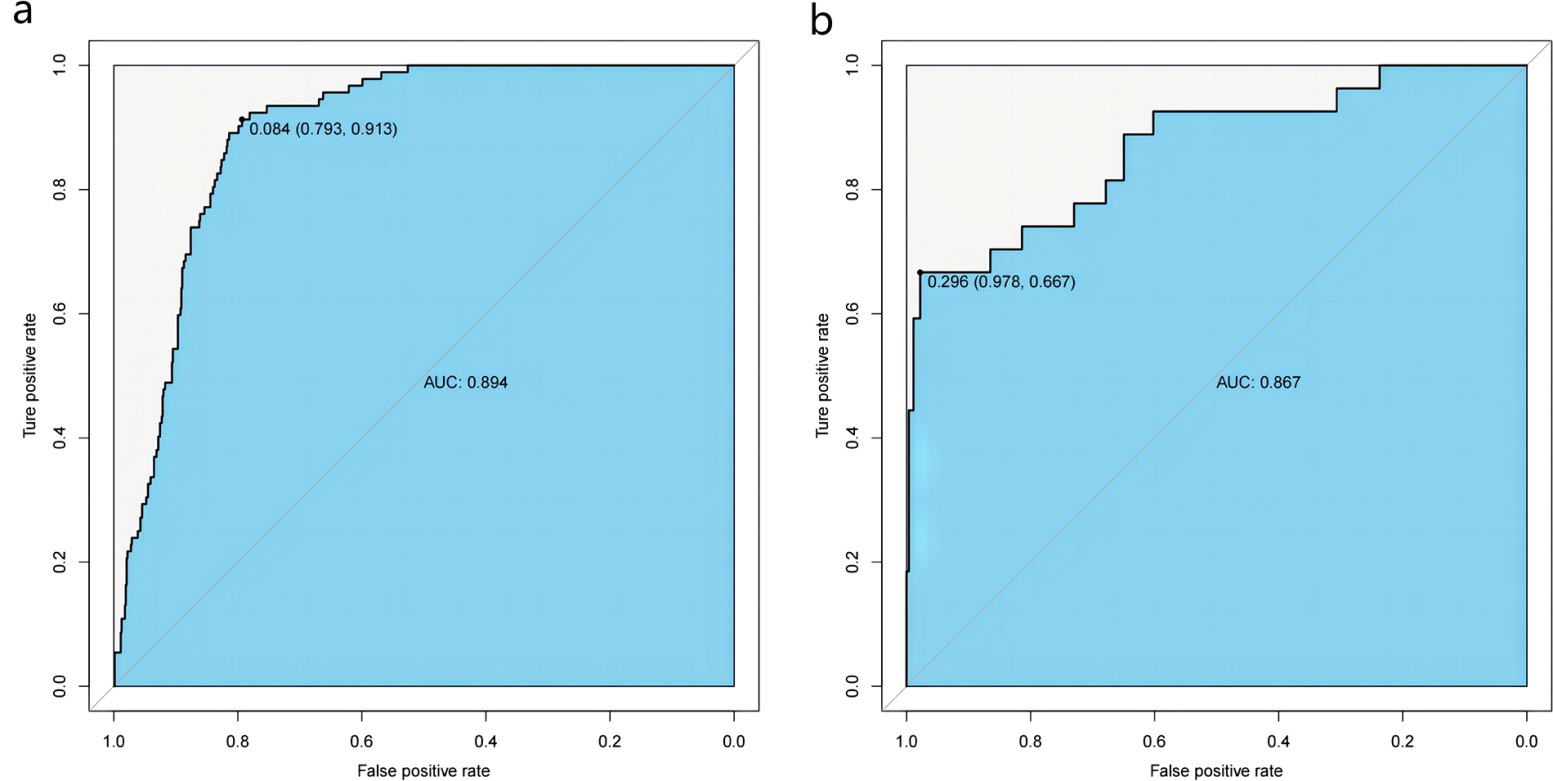
Receiver-operating characteristic (ROC) curves for the nomogram in the training **(a)** and validation sets **(b)** the predictive accuracy of the nomogram was positively correlated with the area under the curve (AUC). The AUC of the nomogram was 0.894 and 0.867 in the training and validation sets, respectively, indicating that the model had good discriminative ability.

**Figure 4:**
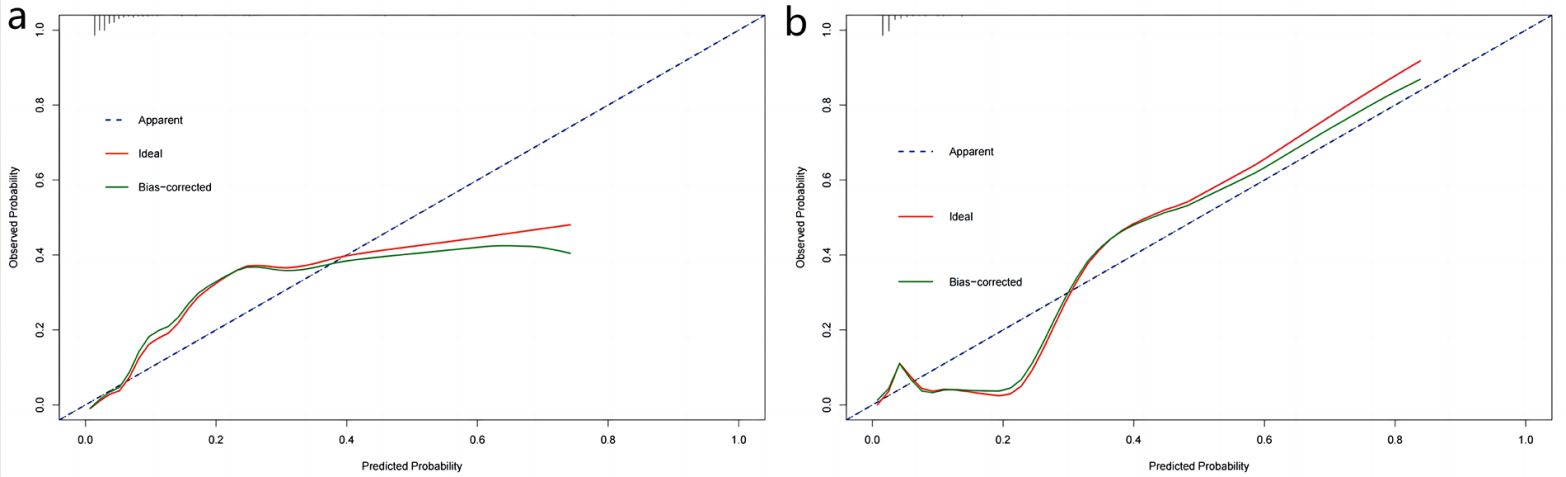
Calibration curves of nomogram in the training set **(a)** and validation set **(b)** X-axis represents the predicted probability of the model and y-axis represents the actual probability. The closer the red and green curves ft the ideal dashed line, the better the predictive consistency of the nomogram.

**Figure 5:**
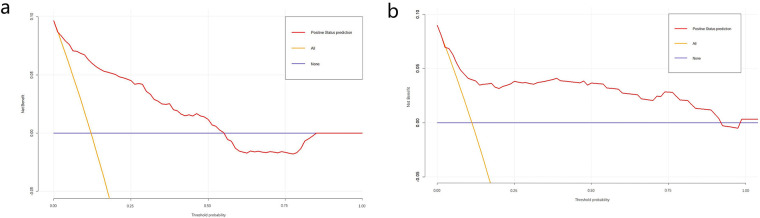
Decision curve analysis (DCA) of nomogram in the training set **(a)** and validation set **(b)** DCA illustrated that the net beneft of the training model is higher in the threshold probability interval of 4%–56%, and the net beneft of the validation model is higher in the threshold probability interval of 5%–92%.

The obtained results were translated into a nomogram designed for predicting the risk of FB (Figure 2). Subsequently, a comprehensive evaluation of the nomogram's performance and reliability was conducted. The AUC for the predictive model demonstrated a robust discriminatory ability at 0.894 (95% CI: 0.8684–0.9188) (Figure 3), with a sensitivity of 91.03% and specificity of 79.3%. These metrics underscore the effectiveness of the model in predicting risk. The nomogram attained a C-index of 0.894, which remained consistent with an adjusted value of 0.883 after 1,000 bootstrap verifications, indicating strong model performance and reliability. In addition, the calibration curves (Figure 4) illustrated the agreement between the predicted and observed probabilities of FBs in trauma patients as per the nomogram. The decision curve analysis (DCA) for the nomogram also reveals a positive net benefit when compared to no intervention, particularly within the threshold probability range of 4%–56% (Figure 5). The findings described herein bolster the practical utility of the FB prediction nomogram in guiding clinical decision-making.

As depicted in Figure 2, a nomogram encompassing five covariates was constructed. To utilize the nomogram effectively: firstly, the subject's BMI (expressed in kg/m^2^) was located on the corresponding variable axis. Then, a vertical line was drawn to intersect with the “Points” axis to obtain the corresponding score. This process was iterated for each covariate, and the scores were cumulatively totaled. The cumulative score was then matched with the corresponding point on the “Total Points” axis, and a vertical line was drawn to intersect with the “Risk of FB” axis to determine the probability of developing FB.

## Discussion

To the present knowledge, this study represents the first attempt to develop and validate a nomogram specifically designed to predict the risk of FBs in patients with postoperative pilon fractures. The present findings reveal an FB incidence of 10.63%, with the multifactorial analysis identifying several independent risk factors for FB, including BMI, fixation mode of fracture, the mode of surgical suture, postoperative infection, NEU, and CKMB. However, in this study, the individualized differences between the two groups (including gender and age) were not found to be statistically significant in univariate analysis (*p* < 0.01).The nomogram, constructed based on these five factors, demonstrated notable performance metrics, with an AUC value of 0.894 (95% CI: 0.8684–0.9188), a sensitivity of 91.03% and specificity of 79.3%. Notably, internal processes underscored the robust consistency of this predictive model.

**Figure 6:**
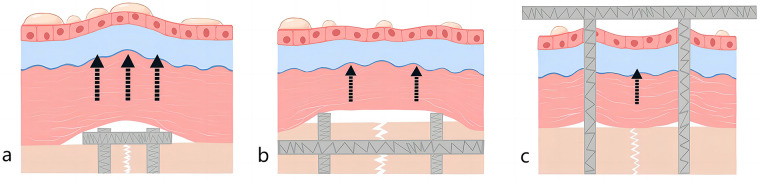
Schematic diagram after pilon fracture **(a)** plate fixation **(b)** intramedullary nail **(c)** external fixation.

Pilon fractures encompass a wide spectrum of injury mechanisms, patient demographics, and both soft-tissue and bone lesions. Patients frequently present with highly comminuted fracture patterns and significant compromise of soft tissues. Surgical intervention must be approached with utmost care, considering the delicate nature of the soft-tissue envelope and employing precise surgical techniques (25). In cases of open fractures, one management option involves early transarticular external fixation followed by delayed internal fixation (26). However, the majority of authors advocate for open reduction and internal fixation (ORIF) as a preferable strategy for managing closed pilon fractures to mitigate the risk of infection (27–29). In recent years, Kuhn et al. designed a novel retrograde tibial nail (RTN), offering a new avenue for minimally invasive treatment of distal tibial fractures (30). Since external fixation and intramedullary nailing typically require less soft-tissue disruption than plating, the incidence of postoperative fracture blisters is low, which is consistent with the results of the present study. Contrastingly, patients undergoing plate fixation typically experience more severe skin and soft tissue damage, leading to an increased risk of postoperative fracture blisters (Figure 6).

While a direct link between BMI and the incidence of FB following surgical procedures may not have been firmly established, other factors such as hypertension, peripheral vascular disease, lymphatic obstruction, and diabetes have been suggested to significantly influence the formation of fracture blisters. These conditions can lead to the impairment of the skin's microvasculature, which may contribute to the development of fracture blisters (31). Notably, BMI is strongly associated with these diseases. Firstly, it has been well-documented in prior research that obese individuals are more likely to experience hypertension compared to those who are not obese (32). Lelong et al. also indicated that the primary objective in addressing the rising prevalence of hypertension in the general population should be the management of weight gain (33). Secondly, Sean P. Heffron et al. suggested that elevated BMI may represent a significant independent risk factor for PAD specifically in women, as opposed to men (34). A recent Mendelian Randomization analysis further supported the notion that increasing BMI serves as an independent risk factor for PAD in both sexes (35). At the same time, Christopher L. Sudduth et al. found that obesity-induced lymphedema is almost universal when BMI is greater than 60 (36, 37). Finally, Natallia Gray et al. discovered that excess weight and obesity significantly contribute to the development of type 2 diabetes mellitus and its associated complications in both males and females (38).

Jialiang Guo et al. proposed that these blisters serve a purpose beyond skin injury, acting as an effective mechanism for pressure release (39). Simultaneously, Varela et al. reported that blister formation serves as one of the mechanisms for alleviating abnormally elevated pressure within a compartment (16). Postoperative infection may result in local tissue edema, which elevates pressure within the fascial compartment and heightens the probability of FBs. Contrastingly, the implementation of extension-reduction stitches following pilon fracture surgery may alleviate pressure within the fascial cavity, consequently diminishing the occurrence of FBs. This explains the lower incidence of postoperative infection and the higher utilization rate of extension-reduction stitches in the non-FB group compared with the FB group.

After direct trauma, the immediate release of cytokines, combined with the immune response, leads to the liberation of a significant quantity of cytokines, as per the conventional understanding. These cytokines subsequently induce a localized inflammatory response and propagate inflammation to the neighboring healthy tissues (13). The present research demonstrates that in the group with blisters, levels of neutrophils and the incidence of postoperative infection were higher compared to those in the group without blisters, aligning with the conventional understanding. At the same time, Yiran Li et al. suggested that FB patients exhibited different cytokine patterns in FB fluid in the early and late stages. Additionally, cytokine patterns in the plasma of FB patients changed as blisters developed. Patients' conditions transitioned gradually from an inflammatory phase to a proliferative repair phase. By monitoring changes in cytokines in FB fluid and plasma, it was observed that different cytokine activity states occurred during different periods. As FBs progressed in development, there was a concurrent alleviation of the systemic inflammatory state, indicating a favorable trend (40). This observation also supports the notion that a considerable portion of the patients with FBs included in the present study were in the inflammatory phase.

CKMB has been extensively adopted to detect acute myocardial injury in clinical practice owing to its high sensitivity and specificity (41). It has also been applied to monitor other injuries, such as skeletal muscle injury (42, 43), pulmonary embolism (44), and brain injury (45). Nonetheless, to the present knowledge, few studies have reported the effects of CKMB on the detection of FB in patients with postoperative pilon fractures. In the present study, its role in the prediction of FB following operation was explored, and findings were made that CKMB played a crucial role in FB according to univariate analysis and logistic regression analysis.

The present study provides significant scientific insights, notably the development and thorough validation of a nomogram. However, it is critical to acknowledge several limitations. Firstly, the present study's retrospective nature inherently introduces the risk of selection bias, which cannot be avoided. Secondly, certain variables that could potentially influence the incidence of FBs were either not recorded or not measured, including the duration of limb immobilization. Thirdly, the clinical data used to develop and validate the nomogram were obtained from a single healthcare center. As such, caution should be exercised when extrapolating the findings to more diverse populations and regions. More data from other healthcare could be collected to verify this model. Emphasizing the need for prospective observational studies incorporating data from multiple centers is essential. This approach would facilitate a more robust assessment of the clinical utility of the proposed model.

Nomograms, widely employed in clinical predictive model studies (46–49), leverage the predictive value of individual risk factors to present final predictions visually. In the present study, a nomogram designed to assist clinicians in evaluating the risk of developing FBs postoperatively in patients recently admitted with pilon fractures was successfully developed and validated. The nomogram incorporates six predictors derived from routinely collected clinical data, accessible within a few hours of emerging FBs. The utilization of the nomogram expedites the identification of patients at an elevated risk of postoperative FBs. Clinicians can readily ascertain the predictive probability for each variable by drawing vertical lines corresponding to the variable outcomes on the nomogram. They can then aggregate these values to calculate the associated risk.

## Conclusion

In conclusion, the present investigation reveals several pivotal factors that serve as significant predictors for FB in individuals afflicted with postoperative pilon fractures. These factors encompass BMI, fixation mode of fracture, the mode of surgical suture, postoperative infection, NEU, and CKMB. To refine the precision of risk assessment, a carefully crafted nomogram was developed based on the identified predictors. This nomogram, subjected to rigorous internal validation, exhibited exceptional discriminatory capability and practical utility in clinical settings. The present examination of the model's performance through validation procedures yielded consistently satisfactory outcomes. The incorporation of the proposed nomogram into clinical practice can provide healthcare professionals with a valuable instrument for promptly assessing the risk of FBs in hospitalized patients, particularly those diagnosed with postoperative pilon fractures.

For patients at high risk of FB, elevation of the affected limb may be taken first. Secondly, ice is applied to the affected area to constrict the blood vessels and reduce the permeability of the blood vessels, thus reducing leakage. Finally, anti-inflammatory drugs can be used to reduce swelling and promote blood circulation. These measures can greatly reduce the risk of postoperative FB, while reducing the risk of infection after rupture, and ultimately improve patient care outcomes.

## Data Availability

The raw data supporting the conclusions of this article will be made available by the authors, without undue reservation.
